# Thyroglobulin Value Predict Iodine-123 Imaging Result in Differentiated Thyroid Cancer Patients

**DOI:** 10.3390/cancers15082242

**Published:** 2023-04-11

**Authors:** Alfredo Campennì, Rosaria Maddalena Ruggeri, Massimiliano Siracusa, Davide Romano, Giulia Giacoppo, Ludovica Crocè, Helena Rosarno, Simona Russo, Davide Cardile, Francesca Capoccetti, Angela Alibrandi, Sergio Baldari, Luca Giovanella

**Affiliations:** 1 Unit of Nuclear Medicine, Department of Biomedical and Dental Sciences and Morpho-Functional Imaging, University of Messina, 98124 Messina, Italy; 2 Unit of Endocrinology, Department of Clinical and Experimental Medicine, University of Messina, 98125 Messina, Italy; 3 Nuclear Medicine Unit, Service Department Macerata Hospital, ASUR Marche AV3, 62100 Macerata, Italy; 4 Unit of Statistical and Mathematical Sciences, Department of Economics, University of Messina, 98125 Messina, Italy; 5 Clinic for Nuclear Medicine and Competence Centre for Thyroid Diseases, Imaging Institute of Southern Switzerland, Ente Ospedaliero Cantonale, 6500 Bellinzona, Switzerland; 6 Clinic for Nuclear Medicine, University Hospital and University of Zurich, 8006 Zurich, Switzerland

**Keywords:** iodine-123 imaging, SPECT/CT, radioiodine diagnostic scintigraphy, differentiated thyroid cancer, thyroglobulin

## Abstract

**Simple Summary:**

This study was prompted to assess the diagnostic performance of ^123^I-Dx-WBS-SPECT/CT in the identification of incomplete structural response in the early follow-up of DTC patients and derived optimized basal-Tg thresholds as a yardstick for scintigraphic imaging. In this light, we reviewed the records of 124 low or intermediate-risk DTC patients who underwent thyroid surgery followed by radioiodine therapy. The response to initial treatments was evaluated 6–12 months after radioiodine therapy. According to 2015 ATA criteria, 87, 19 and 18 patients were classified to have excellent response (ER), indeterminate/incomplete biochemical response (BIndR/BIR) or structural incomplete response (SIR), respectively. Among 37 patients with less than ER, 18 had a positive ^123^I-Dx-WBS-SPECT/CT. Interestingly, metastatic disease noted at ^123^I-Dx-WBS-SPECT/CT mainly involved lymph nodes of the central compartment with a corresponding negative ultrasound of the neck. The optimized basal-Tg cut-off was settled at 0.39 ng/mL by ROC curve analysis (AUC = 0.852) aiming to discriminate patients with positive or negative ^123^I-Dx-WBS-SPECT/CT, respectively. Basal-Tg exceeding this cutoff level independently predicts a positive ^123^I-Dx-WBS-SPECT/CT. In conclusion, ^123^I-WBS-SPECT/CT identified lymph node metastases in 14/37 patients with less than ER and negative neck ultrasound, thus modifying the management of such patients. The diagnostic performance of ^123^I-Dx-WBS-SPECT/CT imaging significantly increases in patients with basal-Tg levels ≥ 0.39 ng/mL.

**Abstract:**

**Background:** In differentiated thyroid cancer (DTC) patients, the response to initial treatments is evaluated 6–12 months after radioiodine therapy (RIT) according to the 2015 American Thyroid Association (2015 ATA) criteria. In selected patients, diagnostic 131-radioiodine whole-body scintigraphy (Dx-WBS) is recommended. We evaluated the diagnostic performance of ^123^I-Dx-WBS-SPECT/CT imaging in detecting incomplete structural responses in the early follow-up of DTC patients and, additionally, derived optimized basal-Tg value as a yardstick for scintigraphic imaging. **Methods:** We reviewed the records of 124 low or intermediate-risk DTC patients with negative anti-thyroglobulin antibody. All patients had undergone (near)-total-thyroidectomy followed by RIT. The response to initial treatments was evaluated 6–12 months after RIT. **Results:** According to the 2015 ATA criteria, 87, 19 and 18 DTC patients were classified to have excellent response (ER), indeterminate/incomplete biochemical response (BIndR/BIR) or structural incomplete response (SIR), respectively. Among patients with less than ER, 18 had a positive ^123^I-Dx-WBS-SPECT/CT. Metastatic disease at ^123^I-Dx-WBS-SPECT/CT mainly involved lymph nodes within the central compartment, and corresponding neck ultrasound examinations were negative. The ROC curve analysis was performed to define the best basal-Tg cut-off (i.e., 0.39 ng/mL; AUC = 0.852) able to discriminate patients with and without positive ^123^I-Dx-WBS-SPECT/CT, respectively. The overall sensitivity, specificity, accuracy, PPV and NPV were 77.8%, 89.6%, 87.9%, 56.0% and 95.9%, respectively. Basal-Tg cut-off was an independent risk factor for having a positive ^123^I-Dx-WBS-SPECT/CT. **Conclusion:** ^123^I-Dx-WBS-SPECT/CT identified lymph node metastases in 14/37 patients with less than ER and a negative neck ultrasound, thus modifying the management of such patients. The diagnostic performance of ^123^I-Dx-WBS-SPECT/CT significantly increased in patients with basal-Tg values ≥ 0.39 ng/mL.

## 1. Introduction

Thyroid cancer represents the most frequent endocrine malignancy [1,2,3,4], with differentiated thyroid cancer (DTC) being the most common subtype (80% and more of all thyroid cancers). The incidence of DTC has been increasing in the last decades with an overall increased annual incidence of about 3%, mainly represented by papillary histotype, small tumors and female patients [5,6,7,8,9,10]. Thyroidectomy followed by risk-adapted ^131^I therapy (RIT) and levothyroxine (LT4) therapy are the standard of care leading to excellent response in more than 80% of patients. Notwithstanding, long-term follow-up is recommended after primary treatment since the risk of persistent or recurrent disease is not negligible, especially among intermediate to high-risk patients [5,11,12,13,14]. According to the 2015 ATA guidelines [15], the response to initial treatments is evaluated at 6–12 months by using basal and/or stimulated serum thyroglobulin (Tg) and neck-ultrasound (nUS). The use of diagnostic whole-body scintigraphy (Dx-WBS) with iodine radioisotopes (^123^I or ^131^I) is suggested in selected DTC patients [5,15,16]. Additional single photon emission computed tomography/computed tomography (SPECT/CT) is recommended in addition to planar imaging whenever possible [16]. Indeed, SPECT/CT is able to: (i) detect a higher number of radioiodine-avid foci than planar imaging; (ii) improve anatomic/topographic localization of radioiodine-avid foci; and (iii) distinguish pathological from aspecific uptakes, respectively. As per consequence, the diagnostic performance of radioiodine imaging significantly increased after the introduction of SPECT/CT in clinical practice, especially in detecting small metastatic lymph nodes within the central compartment [5,15,16,17,18]. The diagnostic performance of radioiodine imaging improves in patients with higher Tg levels (either basal and/or stimulated). Thus, the present study was prompted to assess the diagnostic performance of ^123^I-Dx-WBS-SPECT/CT in detecting structural incomplete response (SIR) at early DTC follow-up. In addition, we assessed the relationship between basal/stimulated Tg levels and ^123^I-Dx-WBS-SPECT/CT results to derive optimized Tg cut-off(s) to select patients for ^123^I-Dx-WBS-SPECT/CT, thus reducing the number of useless studies.

## 2. Patients and Methods

### 2.1. Patients

We retrospectively reviewed the records of 124 consecutive DTC patients [F = 94, M = 30; female to male ratio (F/M) = 3.1:1; mean age ± SD = 47.1 ± 14.1, median age = 46, range = 18–81 years] affected by low [n = 61; F = 49, M = 12; female to male ratio (F/M) = 4.1:1; mean age ± SD = 47.5 ± 13.7, median age= 46, range = 27–78 years] or intermediate [n = 63; F = 45, M = 18; female to male ratio (F/M): 2.5:1; mean age ± SD = 46.7 ± 14.3, median age = 44, range = 18–81 years] risk DTC [pT1-T3, Nx (0/1), Mx] referred to the Nuclear Medicine Unit at “G. Martino” University Hospital in Messina (Italy) from 1 January 2018 to 31 December 2021.

The study was conducted in accordance with the Declaration of Helsinki. In addition, the protocol was approved by the Ethics Committee of the “G. Martino” University Hospital (Project identification code 19/17). All subjects gave informed consent for their inclusion prior to enrollment in the study.

From the present study, patients with (i) positive (i.e., >40 IU/mL, see response assessment section) anti-thyroglobulin antibody (TgAb); (ii) age <18 years; and (iii) poorly-DTC were excluded. Most patients carried papillary thyroid carcinoma (PTC) (n = 112 out of 124, 90.3%; F = 85, M = 27; F/M = 3.1:1; mean age ± SD 46.3 ± 13.6, median = 44.5, range 18–81) while 7 and 5 patients only had a follicular thyroid carcinoma (FTC) (F = 5, M = 2; F/M = 2.5:1; mean age ± SD 57.0 ± 12.2, median = 56, range = 39–74) or a Hurthle cell carcinoma (HC), respectively (F = 4, M = 1; F/M = 4:1; mean age ± SD 52.0 ± 19.7, median = 54, range = 28–75). Among PTC patients, 71 (63.4%) had no aggressive variant (e.g., classic variant) while in 41 (36.6%), an aggressive variant was diagnosed (e.g., sclerosing variant, Tall-cell variant and hob-nail variant).

### 2.2. Methods

All patients had undergone (near)-total-thyroidectomy [(n)-TT] followed by RIT within 3 months. Before RIT (i.e., postoperative period), all patients were assessed by a neck ultrasound (nUS) and laboratory test [1 month after surgery] before performing RIT. Afterward, patients underwent RIT after rhTSH administration according to standard protocol (i.e., intra-muscle injection of 0.9 mg rhTSH daily for 2 consecutive days). Administered ^131^I activities ranged from 2220 to 3700 MBq for ablative purpose and 3700 to 5550 MBq for adjuvant purpose, respectively. A post-therapy whole-body scan with single photon emission tomography-computed tomography (PT-WBS-SPECT/CT) was obtained in all patients (5 to 7 days after RIT), as already described [19,20]. Then, all patients were revaluated 3 months after ^131^I therapy by a clinical examination and laboratory test (TSH, FT4, Tg and TgAb), while a laboratory test and additional nUS and ^123^I-Dx-WBS-SPECT/CT were obtained about 9–12 months later (i.e., response to initial treatments, see specific paragraph). ^123^I-Dx-WBS-SPECT/CT examinations were performed to assess the response to initial treatment in patients fulfilling the 2015 ATA criteria for scintigraphic reassessment, including those with positive PT-WBS-SPECT/CT and/or isthmus topography of malignancy, as already reported [16,21]. Additional diagnostic [e.g., fine needle cytology (FNC) and Tg measurement in the aspirate washout] or therapeutic approaches (e.g., lymph node dissection or ^131^I therapy) were performed according to the patient’s response to initial treatment and the judgement of the attending physician.

The response to initial treatments was assessed 8–12 months after ^131^I therapy by:

(i) Neck-ultrasonography carried out by expert ultrasonographers using high-resolution ultrasound systems (Logiq3 Expert, GE Healthcare, Little Chalfont, United Kingdom or ACUSON 3000 Siemens, Erlangen, Germany) equipped with a high energy linear probe (14 MHz or more);
(ii) Laboratory test. Basal Tg and TgAb values were measured on day 1 (basal), before the first rhTSH administration. Stimulated Tg values were measured on day 3 (early stimulated Tg) and 5 (late stimulated Tg) after the last rhTSH-administration. Serum TSH and TgAb were measured at Core Laboratory “G. Martino” University Hospital in Messina (Italy) by fully automated Access^®^ TSH immunochemiluminescent assay (Beckman Coulter, US; reference range 0.4–4.2 mUI/L) and Elecsys^®^ TgAb chemiluminescence immunoassay (Roche, Switzerland; functional sensitivity 40 IU/mL), respectively. For the purpose of the present study, patients’ sera were frozen at −80 °C and centralized at the Laboratory of Clinical Chemistry and Immunology, Department of Laboratory Medicine, Ente Ospedaliero Cantonale, Bellinzona (Switzerland), and serum Tg was measured on the Kryptor^®^ Compact Plus instrument (BRAHMS Thermo Fisher Scientific, Waltham, MA, USA). The Kryptor^®^ hTg-sensitive assay is calibrated against the BCR^®^ 457 international reference standard and the FS (corresponding to inter-assay imprecision of 20%) has been specified as 0.15 µg/L by the manufacturer (Instructions for Use, ThermoFisher). To conform to the 2015 ATA response criteria, Tg values below 0.20 were transformed in 0.20 ng/mL for statistical analysis.
(iii) ^123^I-Dx-WBS-SPECT/CT was obtained using a double-headed gamma camera (Xeleris, GE Medical System. Chicago, IL, USA; Symens Symbia T-series, Erlangen, Germany) equipped with low-energy high-resolution parallel-hole collimators (HEHRPAR). Whole-body images were obtained from head to proximal thighs (anterior and posterior views, matrix 1024 · 256, magnification: 1, acquisition time: 10 cm/min). The study was integrated by static images of the neck and thorax (anterior and posterior views, magnification: 1; matrix: 256 · 256; frame time: 900 s). SPECT/CT images were obtained in step-and-shoot acquisition mode (40 s/step, 6° angle, 30 steps/detector) while CT images were acquired under the following conditions: tube voltage of 120 kVp, tube current of 80–210 mA, helical thickness of 2.5 mm, table speed of 37 mm/s, table feed per rotation of 18.75 mm/rot, tube rotation time of 0.8 s and pitch of 0.938:1. Using the aforementioned parameters, the overall efficacy doses released to the body linked to the use of ^123^I and CT imaging were 3.145 and <3 mSv, respectively. Finally, all patients were required to drink at least 1.5 L of water and take laxative drugs 1 day before the examination in order to achieve a better target/background ratio.

According to the 2015 ATA guidelines (15), responses to treatment were classified in three categories: (I) excellent response (ER) [i.e., stimulated Tg < 1 ng/mL, and no loco-regional and/or distant metastases at morphological and/or functional imaging; (II) biochemical indeterminate (BIndR) or incomplete (BIR) response [i.e., negative imaging and stimulated Tg between 1 and 10 ng/mL or >10 ng/mL, respectively]; and (III) structural incomplete response (SIR) [evidence of structural disease independently from Tg and TgAb results].

## 3. Statistical Analysis

Continuous data were expressed as mean and standard deviation and categorical variables as numbers ratio and relative percentage. The non-parametric approach was used since most of the numerical variables were not normally distributed, as verified by the Kolmogorov—Smirnov test. In order to assess the existence of significant differences between groups of patients (ER vs. less than ER), the Mann—Whitney test was applied with references to numerical parameters [basal Tg (day 1), early and late stimulated Tg (day 3 and day 5, respectively) and the Chi-Square test for categorical variables. The Spearman correlation test was applied in order to assess the possible correlation between the Tg values (day 1, day 3 and day 5) and ^123^I-Dx-WBS-SPECT/CT results, respectively. The Receiver Operating Characteristic (ROC) curve was plotted in order to set the optimal Tg cut-off for day 1, day 3 and day 5 to predict a positive ^123^I-Dx-WBS-SPECT/CT result; the area under the curve (AUC) was calculated with a relative 95% Confidence Interval and the significance. Sensitivity, specificity, negative predictive value (NPV), positive predictive value (PPV) and diagnostic accuracy (DA) were evaluated according to cut-offs resulting through ROC analysis (0.39, 0.38 and 0.71 for day 1, day 3 and day 5, respectively). Finally, univariate and multivariate logistic regression models were estimated in order to identify significantly independent predictive factors for positive ^123^I-Dx-WBS-SPECT/CT. The covariates were gender, age, histotype, histological variant, size, malignant nodule topography and basal Tg (day 1) cut-off. Statistical analyses were performed using SPSS 27.0 for Window package. A *p*-value lower than 0.05 was considered to be statistically significant.

## 4. Results

Demographic, clinical and pathological data of the enrolled patients are reported in Table 1.

Overall, 87 (70.2%) patients showed an ER (Group A) while the remaining patients (n = 37, 29.8%) had a less than ER. In particular, 19 out of 124 patients (15.3%) were classified to have BIndR [17 (89.5%); median basal Tg (day1) = 0.35 ng/mL, median stimulated Tg (day 5) = 2.9 ng/mL] or BIR [2 (10.5%); median basal Tg (day 1): 1.95 ng/mL, median stimulated Tg (day 5) = 15.9 ng/mL] (Group B), while SIR was diagnosed in 18 patients (14.5%) (Group C). Among these latter patients, seven had shown metastatic disease (lymph node metastases and lymph node and lung metastases in six and one cases, respectively) at ^131^I-PT-WBS-SPECT/CT.

Demographic, clinical and pathological data of the patients with ER, BIndR/BIR and SIR are reported in Table 2, Table 3, Table 4 and Table 5.

Interestingly, among patients of the Group B, no one developed structural disease during the subsequent follow-up. Patients with BIndR did not undergo any treatment (i.e., wait and see strategy) and basal Tg decreased and became undetectable over time with corresponding negative nUS examinations. Conversely, as per local protocols, BIR patients underwent an additional ^131^I therapy course, and relative PT-WBS-SPECT/CT examinations showed radioiodine-avid lymph nodes located in the central compartment. At follow-up evaluation, one of these patients showed an excellent response after retreatment while the remaining one was down-staged from BIR to BIndR status, respectively. It is noteworthy that 123I-Dx-WBS-SPECT/CT was positive (i.e., abnormal foci) in all SIR patients with sensitivity, specificity, positive predictive value (PPV), NPV and accuracy of 48.6%, 100%, 100%, 82.1% and 84.6%, respectively. Metastatic disease detected at 123I-Dx-WBS-SPECT/CT involved local (i.e., VI, VII Robbins’ level), regional (i.e., II–V Robbins’ level) and loco-regional (i.e., VI, IV Robbins’ level) lymph nodes in 13, four and one patients, respectively. Interestingly, lymph node metastases were located within the tracheo-esophageal space in 9 out of 14 patients with local metastatic disease. The overall mean size of the metastatic lymph nodes was 11.1 ± 3.7 mm (median = 11, range = 6–18 mm). Notably, ^123^I-planar imaging detected functional disease in 6 out of 18 patients (sensitivity 33%), confirming the significantly higher sensitivity of SPECT/CT imaging (*p* = 0.006). Notably, nUS was negative in 14 out of 18 patients. In particular, nUS did not detect any local lymph node metastases while pathological lymph nodes were detected in four out of five patients with regional (n = 3) or loco-regional (n = 1) metastatic disease. Accordingly, overall nUS sensitivity, specificity, PPV, NPV and accuracy were 10.8%, 100%, 100%, 72.5% and 73.4%, respectively. However, excluding patients with local lymph node metastases (n = 14), nUS diagnostic performance in detecting regional or loco-regional lymph node metastasis dramatically increased [sensitivity, specificity, positive predictive value (PPV), NPV, accuracy 80%, 100%, 100%, 98.9% and 73.4%, respectively). Regional or loco-regional lymph node metastasis was confirmed in four out of five patients by FNC with Tg washout measurement, and all SIR patients underwent functional lymphadenectomy according to local standard of care (also taking into account that in 9 out of 18 patients, the size of the metastatic lymph node was >10 mm), while no patients accepted a “wait and see” approach. No patients suffered by early or late side effects/complications due to surgical approach while lymph node metastases were confirmed at histopathological analysis in all SIR patients.

Notably, serum Tg values at day 1 (basal measurement), day 3 (early stimulated measurement) and day 5 (late stimulated measurement) were significantly higher in patients with less than ER and positive ^123^I-Dx-WBS-SPECT/CT (median value day 1, day 3 and day 5: 0.97, 3.5 and 7.6 ng/mL, respectively) than those with negative imaging, respectively (median value day 1, day 3 and day 5: 0.7, 2.15 and 3.6 ng/mL, respectively) (*p* = 0.036, *p* = 0.038 and *p* = 0.047, respectively) (Table 6).

**Figure 1:**
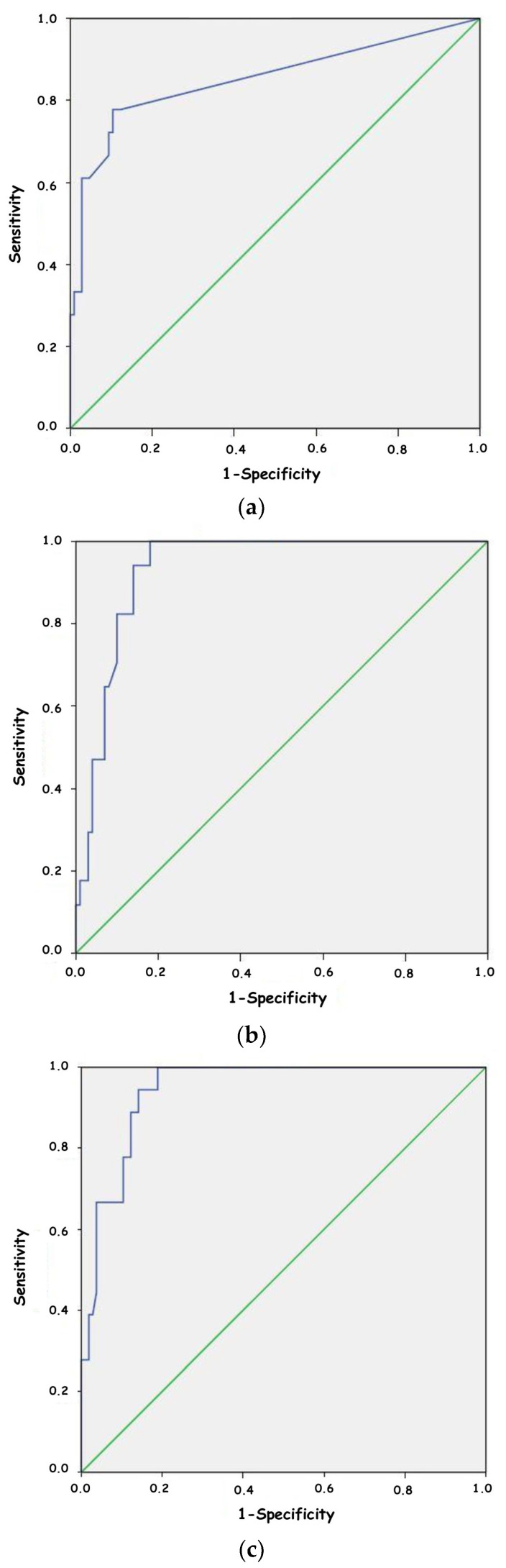
Receiver operating characteristic (ROC) analysis. (**a**) Basal Tg, optimal cutoff = 0.39 ng/mL. Area under the ROC curve (AUC)= 0.852. Sensitivity, specificity, accuracy, positive predictive value (PPV), and negative predictive value (NPV): 77.8%, 89.6%, 87.9%, 56.0% and 95.9%, respectively. (**b**) Early-stimulated Tg, optimal cutoff = 0.38 ng/mL. Area under the ROC curve (AUC) = 0.932. Sensitivity, specificity, accuracy, positive predictive value (PPV), and negative predictive value (NPV): 100%, 82.0%, 84.6%, 48.6% and 100%, respectively. (**c**) Late-stimulated Tg, optimal cutoff = 0.71 ng/mL. Area under the ROC curve (AUC) = 0.944. Sen-sitivity, specificity, accuracy, positive predictive value (PPV), and negative predictive value (NPV): 100%, 81.1%, 83.9%, 47.4% and 100%, respectively.

The optimized serum Tg cut-off points derived by ROC curves to discriminate patients with and without positive 123I-Dx-WBS-SPECT/CT were settled at 0.39 ng/mL (AUC = 0.852), 0.38 ng/mL (AUC = 0.932) and 0.71 ng/mL (AUC = 0.944) for basal (i.e., day 1), early stimulated (i.e., day 3) and late (i.e., day 5) stimulated-Tg values, respectively (Figure 1a–c).

The overall sensitivity, specificity, accuracy, PPV and NPV for basal (i.e., day 1), early (i.e., day 3) and late (i.e., day 5) stimulated Tg were 77.8%, 89.6%, 87.9%, 56.0% and 95.9%; 100%, 82.0%, 84.6%, 48.6% and 100%; and 100%, 81.1%, 83.9%, 47.4% and 100%, respectively. In particular, 14 out of 18 SIR patients (Group C) had basal-Tg value > 0.39 ng/mL, while only 24 out of 106 patients (Group A + B patients) had basal Tg > 0.39 ng/mL (*p* = <0.001).

Positive (+LR) and negative (−LR) likelihood ratios for basal (i.e., day 1), early (i.e., day 3) and late (i.e., day 5) stimulated Tg were 7.49 and 0.25, 5.56 and 0.00 and 5.30 and 0.00, respectively.

Finally, in univariate and multivariate logistic regression analysis, the basal Tg cut-off emerged as the only independent predictor of positive ^123^I-Dx-WBS-SPECT/CT results regardless of age, gender, histotype, histological variant, tumor size, malignant nodule topography or risk classification, according to 2015 ATA (Table 7).

**Figure 2:**
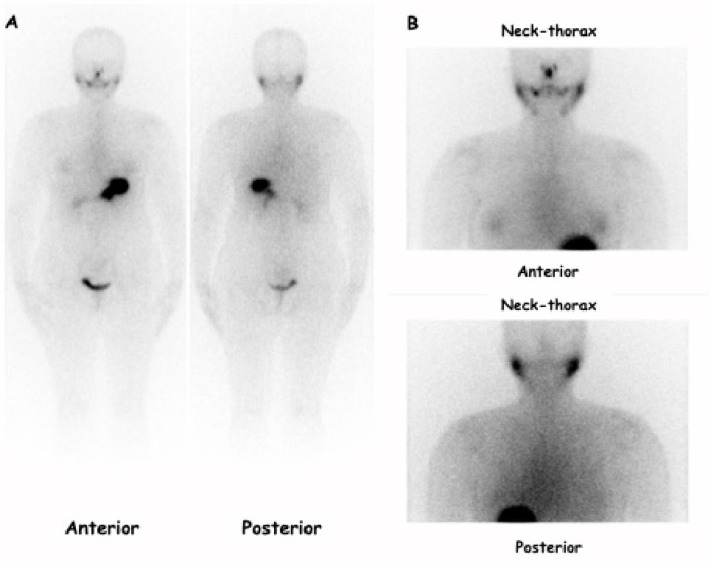

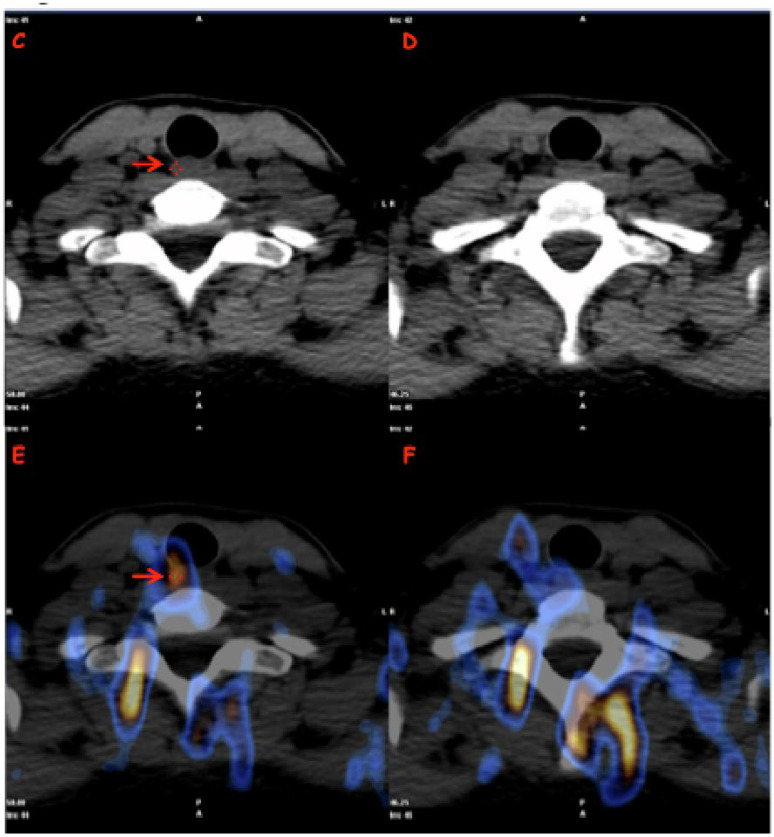
Forty year old woman, pT1b, Nx, Mx-PTC (classic variant), multifocal and bilateral. The largest malignant nodule (14 mm in size) was located in the lower part of the right lobe. (**A**) Dx-WBS (anterior and posterior views) and (**B**) static images (anterior and posterior views) of the neck-thoracic region were obtained 3 h after iodine-123 administration (259 MBq after rhTSH stim-ulation). No pathological radioiodine uptake was noted. On the contrary, axial SPECT-CT images (**C**–**F**) showed abnormal radioiodine uptake in a small para-tracheal lymph-node (red arrows) of the right central compartment (VI Robbins’ level). At the time of 123I-Dx-WBS-SPECT/CT study, nUs did not show any lymph-node with suspected/pathological features. Basal-Tg was 0.9 ng/mL while early and late-stimulated-Tg levels were <10 ng/mL (2.7 and 7.7 ng/mL, respectively). The patient under-went functional lymphadenectomy of the right central compartment and histophatological analysis confirmed the presence of micrometastasis in 4 out of 13 removed lymph-nodes.

Interestingly, the risk of having a positive ^123^I-Dx-WBS-SPECT/CT result was 36% higher in DTC patients with basal Tg ≥ 0.39 ng/mL than others (Odds ratio = 3.60) (Figure 2).

## 5. Discussion

Thyroid surgery followed by postoperative risk-adapted RIT therapy is the standard of care in many DTC patients, leading to an excellent response in more than 80% of patients. The response to initial treatment is commonly assessed 6–12 months after RIT by using a laboratory test (i.e., basal and stimulated Tg measurements) and nUS. In addition, the use of ^131−123^I-Dx-WBS is also recommended in selected DTC patients (e.g., high risk cancers, positive TgAb, poorly informative post-therapy whole-body scintigraphy) according to the 2015 ATA guidelines [15]. More recently, its use has also been suggested in lower risk DTC patients having a malignant nodule located in the isthmus [19,21].

All in all, the use of diagnostic whole-body scintigraphy has markedly decreased in DTC management over the last decades, likely due to suboptimal diagnostic performances in terms of both sensitivity and detection rate. In addition, the so-called stunning effect due to diagnostic ^131^I activity administration further contributed to the reduced use of diagnostic whole-body scintigraphy, since it may reduce the effectiveness of subsequent RIT cycles, as reported by some authors, while others have contradicted this hypothesis [15,22,23,24,25]. Notably, the improved sensitivity of both Tg assays and nUS also contributed to the decreased use of diagnostic whole-body scintigraphy in clinical practice [12,13,25]. All in all, a consensus on the use of diagnostic whole-body scintigraphy after initial RIT has not been reached until now [26,27,28].

However, the aforementioned limits have been overtaken by the availability of both hybrid imaging (i.e., SPECT/CT) and ^123^I (rather than ^131^I), that significantly improved diagnostic performances of diagnostic whole-body scintigraphy, as reported in the literature [11,16,17,18,29,30,31,32,33].

Our present results are well in line with previous ones and showed a significantly better diagnostic performance of hybrid over planar imaging (*p* = 0.006). Indeed, ^123^I planar images were positive in 6 out of 37 patients with less than ER (15.4%) and in 6 out of 18 patients with SIR (33%), respectively.

Moreover, hybrid imaging overperformed nUS in detecting lymph node metastasis within the central neck compartment. Indeed, this is not surprising due to the well-known anatomical limitations of the ultrasound exploration of such a district. As per consequence, it can be difficult for well-trained ultra sonographers to detect lymph node metastasis (especially small sized metastatic disease).

In our cohort of DTC patients with less than ER to initial treatments, ^123^I-Dx-WBS-SPECT/CT changed response classification from BIndR (n = 10) or BIR (n = 4) (better prognosis) to SIR (worse prognosis) in 37.8% of patients with increased Tg and negative nUS. Accordingly, a change in patients’ management was prompted.

On the other hand, negative ^123^I-Dx-WBS-SPECT/CT results occurred in 19 out of 37 DTC patients with BIndR (n = 17) or BIR (n = 2). In such patients, nUS was negative as well. It should be noted that many patients in the BIndR range will spontaneously reach and maintain undetectable basal Tg levels during follow-up, thus making a Tg follow-up the most reasonable approach in such cases [15].

Interestingly, serum Tg values at day 1, day 3 and day 5 were all significantly higher in patients with positive ^123^I-Dx-WBS-SPECT/CT than negative ^123^I-Dx-WBS-SPECT/CT (*p* = 0.036, *p* = 0.038 and *p* = 0.047, respectively).

The most relevant result of our present study has been to set an optimized basal Tg cutoff at 0.39 ng/mL able to provide high diagnostic performance and retain an independent prognostic role, regardless of other routinely considered risk factors such as age, gender, histotype, histological variant, tumor size, malignant nodule topography and risk classification according to 2015 ATA. In addition, the risk of having a positive 123I-Dx-WBS-SPECT/CT result in DTC patients with basal Tg ≥ 0.39 ng/mL was 36% higher than in other ones (Odds ratio = 3.60).

Even if the Tg interpretation criteria are adapted to the methods employed in clinical practice due to the high inter-assay Tg variability, our results are well in line with previous studies where highly sensitive assays were adopted. Interestingly, no structural recurrences were detected over time in patients with basal hsTg values below ~0.3–0.4 μg/L in newly available highly sensitive assays, including the method adopted in our study, and basal hsTg emerged as the only independent predictor of cancer relapse in multivariate analysis, conferring hazard ratios for reduced disease-free survival of 67.94 and 81.61, depending on the assay employed [33,34].

As per consequence, the basal-Tg cut-off value could be useful to address the use of ^123^I-Dx-WBS-SPECT/CT in DTC patients with basal-Tg values ≥ 0.39 ng/mL and rule out such procedure in patients with lower basal-Tg values (NPV 95.9%). Some potential limitations of our study must be also addressed. First, considering the retrospective design, a selection bias cannot be completely excluded. However, the study is not interventional, clinical practice in our centers is homogeneous and strict inclusion criteria were adopted, making a significant effect unlikely. Second, the follow-up length might not be considered sufficient in our patients; nevertheless, some recurrences could turn out positive during lifelong follow-up. Indeed, most relapses occur during early follow-up and significant bias is unlikely. Third, nUS were performed in different services by different operators and this may have reduced the overall accuracy. On the other hand, it should be noted that all ultrasonographers were well trained in DTC patients’ management, and used high standing US machines. Moreover, many metastatic lymph nodes were located in anatomical regions difficult to approach by nUS, such as the central neck compartment.

Finally, the differences between different Tg assays in terms of analytical and clinical performance should be carefully accounted for to correctly use Tg assays in clinical practice [13]. Accordingly, cut-off limits reported in our study should be considered with caution, and their transferability to local settings carefully evaluated.

## 6. Conclusions

^123^I-Dx-WBS is a useful diagnostic tool to integrate Tg measurement and nUS in assessing the response to initial treatments in DTC patients. Planar imaging should be completed by SPECT/CT imaging whenever possible to improve the diagnostic performance of the method, especially in detecting small sized lymph node metastases within anatomical regions (i.e., the central compartment) difficult to explore by nUS. In our series, ^123^I-Dx-WBS-SPECT/CT changed the response assessment (from BIndR/BIR to SIR) and, consequently, the clinical management of more than 35% DTC patients with less than ER to initial treatments. The accuracy of ^123^I-Dx-WBS-SPECT/CT increases when patients are selected based on a basal serum Tg level above 0.39 ng/mL. Thus, the use of basal Tg values is suggested to increase the diagnostic accuracy of ^123^I-Dx-WBS-SPECT/CT and reduce the number of useless radioiodine imaging.

## Figures and Tables

**Table 1:** Demographic, clinical and pathological data of the enrolled DTC patients.

	Patients Number (%)	Female (%)	Male (%)	Median Age (Range)	Median Tumor Size (mm) [Range]	Histological Variant (No Aggressive Variant) [e.g.: * CV; § Minimally Invasive] (%)	Histological Variant (Aggressive Variant) [e.g.: ** FV, # TC, ∞ HV, §§ SV, ¶ HC] (%)	Low-Risk Class (2015 ATA) (%)	Intermediate Risk Class (2015 ATA) (%)
**PTC**	112 (90.3)	85 (75.9)	27 (24.1)	44.5 (19–81)	18.1 (3–39)	71 (63.4)	41 (36.6)	52 (46.4)	60 (53.6)
**FTC**	7 (5.6)	5 (71.4)	2 (28.6)	56 (39–74)	26 (10–38)	7 (100)	0 (0.0)	6 (85.7)	1 (14.3)
**HC**	5 (4.1)	4 (80)	1 (20)	54 (28–75)	33 (25–40)	0 (0.0)	5 (100)	3 (60)	2 (40)
**TOTAL**	124	94 (75.8)	30 (24.2)	46 (19–81)	19.2 (3–40)	78 (62.9)	46 (37.1)	61 (49.2)	63 (50.8)

* Classic variant, § Minimally invasive, ** Follicular variant, # Tall cell, ∞ Hobnail variant, §§ Sclerosing variant, ¶ Hurthle cell.

**Table 2:** Demographic, clinical, pathological and imaging data of the patients with excellent response (ER) to initial treatments (Group A).

	Patients Number (%)	Female (%)	Male (%)	Median Age (Range)	Median Tumor Size (mm) [Range]	Histological Variant: (No Aggressive Variant) [e.g.: * CV; § Minimally Invasive] (%)	Histological Variant (Aggressive Variant) [e.g.: ** FV, # TC, ∞ HV, §§ SV, ¶ HC] (%)	Low-Risk Class (2015 ATA) (%)	Intermediate Risk Class (2015 ATA) (%)	nUS n = Performed Studies [n = Positive studies]	^123^I-Dx-Imaging n = Performed Studies [n = Positive Studies]
**PTC**	77 (88.6)	58 (75.3)	19 (24.7)	48.9 (23–81)	20.5 (3–39)	49 (63.6)	28 (36.4)	36 (46.8)	41 (53.2)	n = 77 [n = 0]	n = 77 [n = 0]
**FTC**	5 (5.7)	4 (80)	1 (20)	49 (39–61)	26 (18–38)	5 (100)	0 (0.0)	4 (80)	1 (20)	n = 5 [n = 0]	n = 5 [n = 0]
**HC**	5 (5.7)	4 (20)	1 (20)	54 (28–75)	33 (25–40)	0 (0.0)	5 (100)	3 (60)	2 (40)	n = 5 [n = 0]	n = 5 [n = 0]
**TOTAL**	87	66 (75.9)	21 (24.1)	49.1 (23–81)	22 (3–40)	54 (62.1)	33 (42.9)	43 (49.4)	44 (50.6)	n = 87 [n = 0]	n = 87 [n = 0]

* Classic variant, § Minimally invasive, ** Follicular variant, # Tall cell, ∞ Hobnail variant, §§ Sclerosing variant, ¶ Hurthle cell.

**Table 3:** Demographic, clinical, pathological and imaging data of the patients with biochemical incomplete/indeterminate response (BIndR/BIR) to initial treatments (Group B).

	Patients Number (%)	Female (%)	Male (%)	Median Age (Range)	Median Tumor Size (mm) [Range]	Histological Variant: (No Aggressive Variant) [e.g.: * CV; § Minimally Invasive] (%)	Histological Variant: (Aggressive Variant) [e.g.: ** FV, # TC, ∞ HV, §§ SV, ¶ HC] (%)	Low-Risk Class (2015 ATA) (%)	Intermediate Risk Class (2015 ATA) (%)	nUS n = Performed Studies [n = Positive Studies]	^123^I-Dx-Imaging n = Performed Studies [n = Positive Studies]
**PTC**	18 (94.7)	15 (83.3)	3 (16.7)	38 (18–50)	15 (5–38)	10 (55.6)	8 (44.4)	6	12	n = 18 [n = 0]	n = 18 [n = 0]
**FTC**	1 (5.3)	0 (0)	1 (100)			1 (100)	0 (0)	1 (100)	0 (0)	n = 1 [n = 0]	n = 1 [n = 0]
**TOTAL**	19	15 (78.9)	4 (21.1)	38 (18–50)	15 (5–38)	11 (57.9)	8 (42.1)	7 (36.8)	12 (63.2)	n = 19 [n = 0]	n = 19 [n = 0]

* Classic variant, § Minimally invasive, ** Follicular variant, # Tall cell, ∞ Hobnail variant, §§ Sclerosing variant, ¶ Hurthle cell.

**Table 4:** Demographic, clinical, pathological and imaging data of the patients with structural incomplete response (SIR) to initial treatments (Group C).

	Patients Number (%)	Female (%)	Male (%)	Median Age (Range)	Median Tumor Size (mm) [Range]	Histological Variant: (No Aggressive Variant) [e.g.: * CV; § Minimally Invasive] (%)	Histological Variant (Aggressive Variant) [e.g.: ** FV, # TC, ∞ HV, §§ SV, ¶ HC] (%)	Low-Risk Class (2015 ATA) (%)	Intermediate Risk Class (2015 ATA) (%)	nUS n = Performed Studies [n = Positive Studies]	^123^I-Dx-Imaging n = Performed Studies [n = Positive Studies]
**PTC**	17 (94.4)	12 (70.6)	5 (29.4)	42 (24–72)	12 (6–24)	11 (64.7)	6 (35.3)	10 (58.8)	7 (41.2)	n = 17 [n = 4]	n = 17 [n = 17]
**FTC**	1 (5.6)	1 (100)	0 (0)			1 (100)	0 (0)	1 (100)	0 (0)	n = 1 [n = 0]	n = 1 [n = 1]
**TOTAL**	18	13 (72.2)	5 (27.8)	42 (24–72)	12 (6–24)	12 (66.7)	6 (33.3)	11 (61.1)	7 (38.9)	n = 18 [n = 4]	n = 18 [n = 18]

* Classic variant, § Minimally invasive, ** Follicular variant, # Tall cell, ∞ Hobnail variant, §§ Sclerosing variant, ¶ Hurthle Cell.

**Table 5:** Comparison of clinical characteristics of the patients included in different groups.

	Patients Number (%)	Female (%)	Male (%)	Median Age (year)	PTC (%)	FTC (%)	Median Tumor Size (mm)	Histological Variant: (No Aggressive Variant) [e.g.: * CV; § Minimally Invasive] (%)	Histological Variant (Aggressive Variant) [e.g.: ** FV, # TC, ∞ HV, §§ SV, ¶ HC] (%)	Low-Risk Class (2015 ATA) (%)	Intermediate Risk Class (2015 ATA) (%)
**Group A**	87 (70.2)	66 (75.9)	21 (24.1)	49.1	77 (88.5)	10 (11.5)	22	54 (62.1)	33 (42.9)	43 (49.4)	44 (50.6)
**Group B**	19 (15.3)	15 (78.9)	4 (21.1)	38	18 (94.7)	1 (5.3)	15	11 (57.9)	8 (42.1)	7 (36.8)	12 (63.2)
**Group C**	18 (14.5)	13 (72.2)	5 (27.8)	42	17 (94.4)	1 (5.6)	12	12 (66.7)	6 (33.3)	11 (61.1)	7 (38.9)

* Classic variant, § Minimally invasive, ** Follicular variant, # Tall cell, ∞ Hobnail variant, §§ Sclerosing variant, ¶ Hurthle cell.

**Table 6:** Basal, Early and late-stimulated-Tg values of all DTC patients.

	Basal-Tg (Day 1)		Early-Stimulated-Tg (Day 3)		Late-Stimulated-Tg (Day 5)	
	Median (ng/mL)	Range (ng/mL)	Median (ng/mL)	Range (ng/mL)	Median (ng/mL)	Range (ng/mL)
**Group A** **(n = 87)**	0.15	0.15–0.51	0.15	0.15–0.83	0.15	0.15–0.67
**Group B** **(n = 19)**	0.70	0.15–2.1	2.15	0.15–16.9	3.6	0.9–19.8
**Group C** **(n = 18)**	0.97	0.15–11.4	3.50	0.42–47.3	7.6	0.76–41
**All patients** **(n = 124)**	0.15	0.15–11.4	0.15	0.15–47.3	0.15	0.15–41

**Table 7:** Univariate and multivariate logistic regression analysis for positive ^123^I-Dx-imaging after initial treatments: basal-Tg ≥ 0.39 ng/mL.

Independent Variables	Univariate Models			Multivariate Model		
	OR	95% CI	*p*-Value	OR	95% CI	*p*-Value
**Age**	0.997	0.962–1.033	0.866	1.000	0.953–1.049	0.994
**Gender**	0.802	0.261–2.471	0.701	1.045	0.223–4.902	0.955
**Histotype**	0.508	0.062–4.195	0.530	0.212	0.008–5.338	0.346
Histological variant *	0.830	0.299–2.308	0.721	1.594	0.362–7.019	0.538
**Basal-Tg** **(≥0.39 ng/mL)**	30.227	8.450–108.123	**<0.001**	34.625	8.359–143.421	**<0.001**
**Tumor size**	0.964	0.917–1.012	0.142	0.979	0.925–1.036	0.460
**Risk classification #** **(2015 ATA)**	0.568	0.205–1.578	0.278	0.406	0.105–1.570	0.192
**Malignant nodule topography**	0.980	0.111–8.660	0.986	0.634	0.035–11.401	0.757

Abbreviations: OR, Odds Ratio; CI, Confidence Interval; * No aggressive variant: Classic variant, Minimally invasive tumor; Aggressive variant: Follicular variant, Tall-cell variant, Hobnail variant, Sclerosing variant, Hurthle cell. # Low-risk cancer; Intermediate risk cancer.

## Data Availability

The data that support the findings of this study are available on request from the corresponding author [A.C.].
